# Quantitative Sensory Testing in a Girl With Tangier Disease: A Case Report

**DOI:** 10.7759/cureus.81676

**Published:** 2025-04-03

**Authors:** Edicson Ruiz-Ospina, Fernando Ortiz-Corredor, Sandra Milena Castellar-Leones, Javier Rubio Delgado-Martinez

**Affiliations:** 1 Rehabilitation Medicine, Universidad Nacional de Colombia, Bogotá, COL; 2 Rehabilitation Medicine, Universidad Nacional de Colombia, San Juan de Pasto, COL

**Keywords:** children, neuropathic disorders, quantitative sensory test, small fiber neuropathy, tangier's

## Abstract

Tangier disease is a rare genetic disorder caused by a deficiency in the ABCA1 gene, resulting in impaired metabolism of high-density lipoprotein (HDL) cholesterol. This disorder affects multiple organs, including the nervous system, and presents with a range of neurological symptoms. The primary neurological manifestation is peripheral nerve dysfunction, characterized by numbness, tingling, and weakness in the extremities. Autonomic dysfunction may also occur, causing bowel and bladder abnormalities. Cholesterol accumulation in nerve tissues is believed to be responsible for these symptoms.

Research has shown that patients with Tangier disease commonly exhibit electrophysiological characteristics indicative of large fiber neuropathy. Small fiber neuropathy, a condition involving damage to small, unmyelinated nerve fibers, is not typically considered a defining feature of the disease. However, diagnostic methods such as quantitative sensory studies, which could help detect small fiber neuropathy, are often overlooked in the literature.

In many cases, the diagnostic approach to neuropathy halts when electrodiagnostic studies return normal results, often disregarding the possibility of an underlying small fiber neuropathy. This may contribute to the underrecognition of small fiber neuropathy in Tangier disease.

## Introduction

Tangier disease, also known as familial alpha-lipoprotein deficiency, is a rare genetic disorder characterized by the absence of high-density lipoproteins (HDLs) in the blood [1]. This condition is inherited in an autosomal recessive pattern. The gene associated with Tangier disease, called the ATP-binding cassette transporter A1 gene (ABCA1), plays a crucial role in the cellular pathway responsible for the secretion of cholesterol and phospholipids to lipid-poor apolipoproteins [2]. This suggests that when newly synthesized apolipoproteins are unable to acquire cellular lipids through the ABCA1 pathway, they undergo rapid degradation, leading to an over-accumulation of cholesterol within macrophages [3]. Tangier disease is extremely rare, with only a few hundred cases reported worldwide. It appears to affect men and women in equal numbers, and its prevalence varies across different populations [4].

While the symptoms of Tangier disease can vary from person to person, they often include an enlarged liver and spleen (hepatosplenomegaly), as well as a decrease in the levels of HDL cholesterol in the blood [5]. This condition can also lead to the development of yellowish-orange tonsils and the formation of fatty deposits, known as xanthomas, in various parts of the body as well as peripheral neuropathy [6]. In addition to these physical symptoms, individuals with Tangier disease may also experience cardiovascular complications, such as an increased risk of atherosclerosis and coronary artery disease [7].

Diagnosis of Tangier disease typically involves a combination of clinical evaluation, laboratory tests, and genetic testing. Laboratory tests often focus on lipid measurements, including HDL levels and cholesterol distribution within lipoproteins. Genetic testing can identify mutations in the ABCA1 gene [8]. In addition to these diagnostic measures, imaging studies, such as ultrasound or MRI, may be used to assess the size and condition of the liver and spleen, as well as detect any signs of atherosclerosis [9].

Current treatment options for Tangier disease primarily focus on managing the symptoms and complications associated with the condition. This may involve dietary modifications to minimize cholesterol intake, as well as medications to control lipid levels and reduce the risk of cardiovascular complications [10]. Furthermore, individuals with Tangier disease may benefit from regular monitoring by a multidisciplinary team of healthcare professionals to address the diverse challenges posed by the disorder [11]. Innovative research into potential gene therapy approaches and other targeted interventions holds promise for advancing the treatment of Tangier disease [8]. By gaining a deeper understanding of the underlying molecular mechanisms and genetic pathways involved in the condition, researchers aim to develop more effective strategies to alleviate its impact on affected individuals.

Peripheral neuropathy in Tangier disease can vary widely in presentation. It has been categorized into three major subtypes [12]: (1) syringomyelia-like neuropathy, which is more common and involves a slow, progressive weakness and wasting of the distal upper extremity muscles as well as loss of pain and temperature sensation, typically sparing the lower extremities; (2) multifocal mono- or polyneuropathy, which presents with a multifocal pattern of relapsing-remitting motor and sensory loss; and (3) subclinical or distal symmetric polyneuropathies, which are less common.

Electrodiagnostic studies of peripheral neuropathy in the condition often show demyelinating abnormalities more predominantly in the upper extremities. Moreover, there may be a slowing of motor nerve conduction more prominent in the intermediate nerve segments than in the distal segments [13].

Published case reports have used clinical methods and electrophysiologic studies to demonstrate peripheral neuropathy [14,15]. In addition, sural nerve biopsy, high-resolution ultrasound of the peripheral nerve, and MRI have been used [16]. Sympathetic skin response and RR interval were used to assess small fiber damage [12].

Here, we present the case of a child with Tangier disease in whom peripheral neuropathy was demonstrated using quantitative sensory testing (QST).

## Case presentation

A 12-year-old girl has been experiencing clinical manifestations since the age of five, including recurring episodes of lymphadenopathy in her neck. At the age of nine, she began experiencing abdominal pain associated with mesenteric lymphadenopathy. Later, she began experiencing decreased sensation in her hands and feet, which then spread to all her limbs distal to the elbows and knees. She also experienced generalized pain with an intensity of 8/10 on a visual analog scale and required hospitalization several times for multiple pain crises that necessitated parenteral analgesia. Physical examination revealed palpable hepatosplenomegaly, and her laboratory results indicated an HDL level of 0.9 mg/dl (normal values > 45 mg/dL) and thrombocytopenia.

Whole-exome sequencing of the patient identified two compound heterozygous mutations in the ABCA1 gene: NM_005502.4:c.[16C>T];[5520del] and NP_005493.2:p.[(Gln6Ter)];[(p.Phe1840LeufsTer30)]. The segregation of the variants was confirmed by Sanger DNA sequencing in available family members. These variants have been reported previously and classified as pathogenic in the ClinVar database (https://www.ncbi.nlm.nih.gov/clinvar; variation ID: 1456561 and 2735311)

Symptomatic treatment and dietary management were provided to the patient, and clinical appointments with the interdisciplinary team were initiated.

After being discharged from the hospital, the patient was in good general physical condition and independent in all her self-care activities. She had a normal gait and was able to climb the stairs, run, and jump. However, she continued to experience generalized pain in her extremities, characterized by numbness, tingling, and occasional cooling. She was treated with a combination of gabapentin and acetaminophen.

Functional assessments included the following: Timed Up and Go Test, 7.47 s (reference value: <13 s); Box and Block Test, 60 for the right (R) hand and 56 for the left (L) hand (normal values: R, 60-80; L, 57-80); Five Times Sit to Stand Test, 10 s (normal value: <11 s); Unipedal Stance Test, >30 s for both sides (normal value: >30 s), and Six-Minute Walk Test, 564 m (normal range: 576-700 m).

The QST was conducted in the upper and lower limbs, utilizing a four-, two-, and one-stepping algorithm to estimate the cutaneous sensation threshold. The CASE IV lab software (computer-aided sensory evaluator) was used to evaluate the vibratory and thermal modalities. The stimuli were generated by the system, and the patient was cued to respond. The subject's responses were recorded to determine the sensory threshold. This assessment was conducted with the WR Medical Electronics Co. equipment (Stillwater, MN, USA).

To evaluate the heat pain modality, a non-repeating with null stimuli algorithm was utilized. The vibratory stimulator was set to generate vibrations at 125 Hz, with the amplitude controlled by the computer to range from 0.1 to 576 µm in 25 levels, or just noticeable difference (JND). For the thermal modalities, the temperature was used as the baseline. The computer controlled the thermode's temperature to range from 5 °C to 50 °C, with the stimulus intensity given in 25 levels or JNDs. The rate of temperature change was set at 4 °C/s. The maximum temperatures that could be attained were 9 °C × 10 s for cold perception threshold and 49 °C × 10 s for heat pain perception. CASE IV software calculates the obtained values based on genre and age-matched normograms, providing percentiles and standard deviation to determine thresholds. 

**Figure 1:**
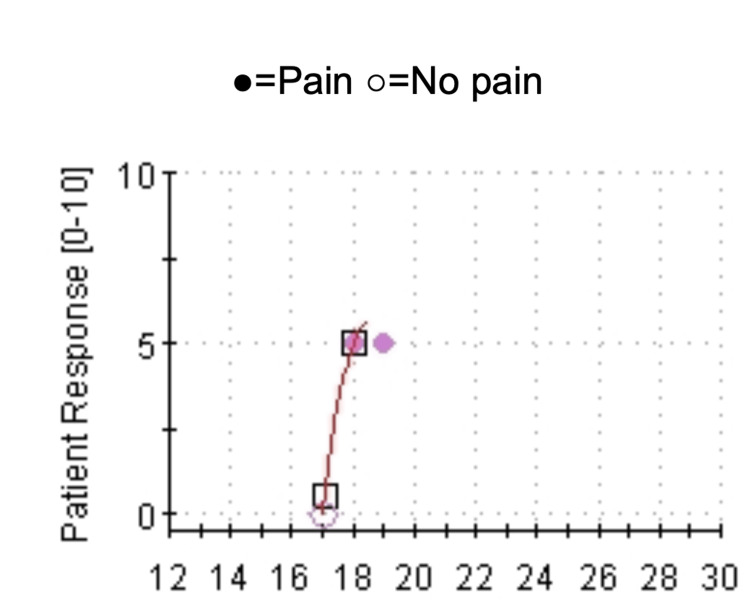
Heat-pain NRA-NS test An increase in thermal pain sensitivity was observed in the dorsum of the right hand. NRA-NS: non-repeating ascending with null stimuli.

The study revealed increased thermal pain sensitivity with percentiles lower than 2.5, indicating allodynia and hyperalgesia (Figure 1).

**Figure 2:**
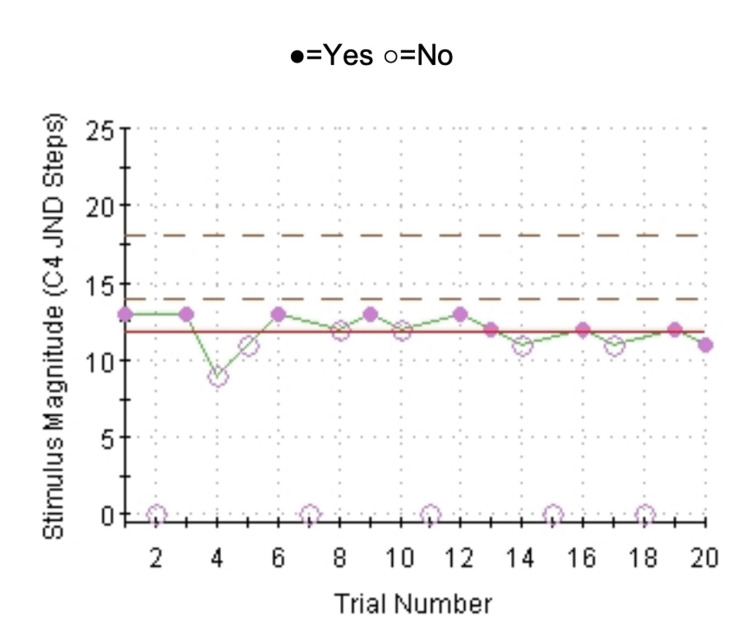
Cold detection: four-, two-, and one-stepping algorithm with NS test Threshold in the left arm showing hyposensitivity to cold stimuli. NS: null stimuli.

**Figure 3:**
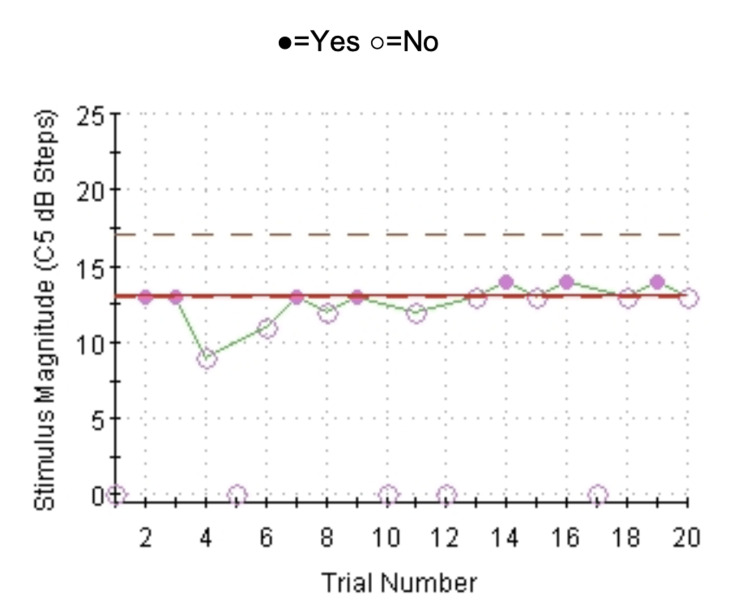
Vibration detection: four-, two-, and one-stepping algorithm with NS test Threshold in the right foot showing hyposensitivity to vibratory stimuli. NS: null stimuli.

Cold and vibratory perception thresholds were above the 97.5 percentile (Figure 2 and Figure 3), suggesting hyposensitivity to cold and vibratory stimuli.

These findings suggest dysfunction of both small and large myelinated nerve fibers (Table 1).

**Table 1:** Quantitative sensory testing results in percentile RUP: right upper limb, LUP: left upper limb, RLL: right lower limb, LLL: left upper limb, NR: not registered.

Limb	Sensitivity		
	Cooling	Heat-Pain	Vibration
RUP	NR	2	99.99
LUP	98	0.29	NR
RLL	97	6	99.99
LLL	97	0.04	99.99

## Discussion

QST is a neurophysiological method that evaluates not only the function of large nerve fibers (Aβ) and the lemniscal system but also the nociceptive and non-nociceptive small nerve fibers (Aδ, C) and the spinothalamic pathways, which are involved in both peripheral and central pain syndromes.

In the presented patient, no muscle weakness or atrophy was observed. However, the patient's burning pain with a generalized distribution suggests small fiber damage, which was confirmed by QST, demonstrating altered sensitivity to pain and temperature. 

QST has been shown to provide reliable results in children aged six years and older and has been used to evaluate pediatric patients with diabetes, familial dysautonomia, complex regional pain syndrome, and sickle cell disease [17]. Reference values for QST in children have been published [18]. Various protocols and equipment are available for QST application in this age group.

Burning pain in the lower limbs has been reported since the initial descriptions of Tangier disease, but detailed evaluations of peripheral nerves and pain in affected children remain limited. Negi et al. described a 38-year-old patient with episodes of ataxia, nystagmus, and strabismus since the age of eight, who had previously been diagnosed with pressure neuropathy of the median and ulnar nerves [19]. Hovingh et al. reported a 38-year-old patient with severe atherosclerosis in the coronary arteries, splenomegaly, corneal opacities, and peripheral neuropathy, who was initially misdiagnosed with Charcot-Marie-Tooth disease in adolescence [20]. In these cases, the presence of pain was not mentioned.

In contrast, Per et al. described a 13-year-old patient in whom neuropathy was the initial clinical manifestation of Tangier disease, beginning with generalized weakness and pes cavus [16]. Electrophysiological signs of axonal polyneuropathy were observed [16]. Although the report does not describe the presence of pain in this patient, the elevated threshold for proprioceptive sensitivity, as revealed by QST, suggests that fast-conducting fibers may also be affected.

In our patient, the probable lack of prominent large fiber involvement may be due to a preclinical state of large fiber neuropathy. However, the elevated threshold for proprioceptive sensitivity, as revealed by QST, suggests that fast-conducting fibers could also be affected in the early phase. Further studies examining phenotype-genotype correlations in Tangier disease related to peripheral neuropathy are needed to determine whether certain variants follow different trajectories, starting with small fiber neuropathy and progressing to established large fiber neuropathy in later stages.

## Conclusions

Neuropathic pain may manifest in children with Tangier disease. QST can be a valuable diagnostic tool for peripheral neuropathy, even in rare diseases like Tangier disease, particularly when combined with a comprehensive clinical examination. Further studies are needed to better understand the sensory profile in children with Tangier disease and the role of QST in identifying small fiber neuropathy or sensory alterations in this patient group. Future implications include evaluating QST as a potential biomarker in the preclinical stages of the disease, as well as for predicting the onset of neuropathic pain and guiding response to neuromodulatory treatments as a strategy for managing Tangier disease.
